# A prospective study of knee pain, low back pain, and risk of dementia: the JAGES project

**DOI:** 10.1038/s41598-019-47005-x

**Published:** 2019-07-23

**Authors:** Keiko Yamada, Yasuhiko Kubota, Takahiro Tabuchi, Kokoro Shirai, Hiroyasu Iso, Naoki Kondo, Katsunori Kondo

**Affiliations:** 1 Public Health, Department of Social Medicine, Osaka University Graduate School of Medicine, Suita, Japan; 2 Department of Anesthesiology and Pain Medicine, Juntendo University Faculty of Medicine, Tokyo, Japan; 3 Department of Psychology, McGill University, Montreal, Canada; 4 Osaka Center for Cancer and Cardiovascular Diseases Prevention, Osaka, Japan; 5 Cancer Control Center, Osaka International Cancer Institute, Osaka, Japan; 6 Departments of Health Education and Health Sociology, The School of Public Health, The University of Tokyo, Tokyo, Japan; 7 Department of Social Preventive Medical Sciences, Center for Preventive Medical Sciences, Chiba University, Chiba, Japan

**Keywords:** Dementia, Musculoskeletal system, Risk factors

## Abstract

The aim of this prospective study was to investigate the associations of knee and low back pain with dementia development. Participants were 14,627 older people with no history of stroke, cancer, injuries, depression, Parkinson’s disease, or dementia who did not require support for daily living completed self-administered questionnaires with 3-years follow-up. A Cox regression model was used to calculate hazard ratios (HRs) and 95% confidence intervals (CIs) for dementia development. Stratified analyses by age and regular walking were conducted. Dementia risk was higher in participants aged 65–79 years with knee pain and without low back pain than in those without knee and low back pain [HR: 1.73 (95% CI: 1.11–2.68)]. Dementia risk was lower in participants ≥80 years with low back pain but no knee pain than in those without low back or knee pain [HR: 0.50 (95% CI: 0.31–0.80)]. Participants with knee pain who did not walk regularly had the highest dementia risk [HR: 1.71 (95% CI: 1.26–2.33)]. Knee pain may increase dementia risk among individuals aged 65–79 years, and may further increase risk in non-regular walkers. Low back pain may be a marker of maintained cognitive function despite age for individuals ≥80 years.

## Introduction

Pain is a major cause of reduced activities of daily living (ADL)^1^, which can increase dementia risk among older people. Thus, pain may be a dementia risk factor. However, previous study findings on pain and dementia are inconsistent. A prospective cohort study in the United States identified persistent pain as a risk factor for cognitive impairment and dementia^2^, whereas pain was not associated with cognitive decline among older people in a prospective cohort study in the United Kingdom^3^.

To explain this inconsistency, it may be useful to consider pain sites. Although both knee pain and low back pain can reduce ADL, they may differentially affect cognitive functions, and an investigation of the associations between knee/low back pain and dementia risk may be useful for dementia prevention. The only previous study on pain sites reported that osteoarthritis (OA) was independently associated with increased risk of dementia development^4^. However, the study did not consider physical activities or psychosocial factors, which are important for dementia development. Furthermore, no studies have investigated the association between low back pain and dementia risk.

Therefore, the aim of this Japanese prospective cohort study was to examine the associations of knee pain and low back pain with dementia development, considering physical activities, psychosocial factors, and differences in underlying mechanisms of knee/low back pain.

## Methods

### Study population

We mailed self-administered questionnaires to older residents (aged ≥65 years) identified from official residential registers of 30 local governments throughout Japan at baseline in 2013. The questionnaires assessed the experience of knee pain and low back pain. Participation was voluntary. The response rate was 71.5%, which is comparable to other surveys of community-dwelling residents. Respondents were linked to the national long-term care insurance registry, which includes information about cognitive impairment based on in-home assessment by trained investigators (e.g. public health nurses)^5^. Under the Long-Term Care Insurance Act, all local governments in Japan retain data on cognitive impairment for all applicants of the national long-term care program.

We identified 17,290 participants who completed the questionnaires and did not received benefits from the national long-term care insurance at baseline. We excluded 1,745 participants with a history of stroke, cancer, injuries, depression, or Parkinson’s disease, dementia at baseline, and we also excluded 918 participants who reported a need for support for daily living or who had missing ADL data. Data from 14,627 participants were analysed. None of the participants had a documented disability.

### Knee pain and low back pain

We used two simple sequential yes/no questions to detect knee pain and low back pain.

#### Knee pain

“Have you had any pain around your knee during the last year? (yes)” and “Has your knee pain interfered with your daily activities? (yes)” defined knee pain.

#### Low back pain

“Have you had any pain around your low back during the last year? (yes)” and “Has your low back pain interfered with your daily activities? (yes)” defined low back pain.

### Adjusted variables

#### Demographic factors

These were age (65–69, 70–74, 75–79, 80–84, or ≥85 years), sex, body mass index (10 percentiles), alcohol consumption (non-drinker, ex-drinker, or drinker), smoking (non-smoker, ex-smoker, or smoker), and history of diabetes mellitus and hypertension (yes or no).

The following single questions were used to measure drinking and smoking status: “Do you drink alcohol? –‘Yes’, ‘Ceased drinking’, or ‘No’” and “Do you smoke? –‘Yes’, ‘Ceased smoking’, or ‘No’.”

#### Socioeconomic factors

Education (<6 years, 6 to <9 years, 9 to <12 years, or ≥12 years), marital status (married, divorced, single, widowed, or other), income divided into quartiles of equivalised income in 2012, including tax: Q1 (men <1.31; women <1.23), Q2 (men 1.31–1.93; women 1.23–1.70), Q3 (men 1.94–2.46; women 1.71–2.46), Q4 (men 2.47–3.19; women 2.47–3.20), and Q5 (men >3.19; women >3.20) million Japanese yen [JPY]), employment status (employee, retired, or unemployed), loss events (loss of spouse, family member, close friend, or relative; yes or no), frequency of social interaction (≥4 per week, 2–3 per week, once a week, 1–3 per month, several times per year, or never), and frequency of going out (≥4 per week, 2–3 per week, once a week, 1–3 per month, several times per year, or never). Equivalised income was calculated by dividing the median value of the multiple-choice annual household income by the square root of the number of people living together. The annual household income question had 15 categories (<0.5, 0.5–0.9, 1–1.4, 1.5–1.9, 2.0–2.4, 2.5–2.9, 3.0–3.9, 4.0–4.9, 5.0–5.9, 6.0–6.9, 7.0–7.9, 8.0–8.9, 9.0–9.9, 10.0–11.9, and >11.9 million JPY). We defined low income as less than 1.22 million JPY of equivalised income above the poverty line in Japan in 2015^6^.

#### Psychological factor

Mood or anxiety disorder (yes or no). When participants had 13 points or more in the Kessler Psychological Distress Scale (K6), they were considered to have mood or anxiety disorder^7^.

#### Others

We additionally adjusted for knee pain or low back pain when we used low back pain or knee pain as a main exposure, respectively.

### Definition of dementia development

We used data from a standardized in-home assessment carried out under the national long-term care insurance scheme established in 2000^5^. The primary assessment was conducted for applicants of the long-term care program by trained investigators dispatched from the certification committee in each municipality. During home visits, each applicant was assessed on their ADL and instrumental ADL status, cognitive function (e.g. short-term memory, orientation, and communication) and the presence of mental and behavioural disorders using a standardized government assessment manual^8^.

Cognitive disability grade was categorized into eight levels: 0, I, IIa, IIb, IIIa, IIIb, IV, and M (0 = Independent, M = Needs constant treatment in a specialized medical facility). This cognitive impairment categorization strongly correlated with Mini-Mental State Examination scores (Spearman’s rank correlation r = −0.73, p < 0.001)^9^, and ‘level I’ corresponded with a 0.5 point rating on the Clinical Dementia Rating scale (specificity and sensitivity, 0.88)^10^.

The certification committee also asked a panel of physicians to independently assess the applicants’ cognitive disability levels to determine the applicants’ care requirements^11^. The medical assessment was conducted independently of the in-home assessment^11^. In our analysis, we used the in-home assessment, but a previous study using some of the JAGES data found a high correlation between in-home assessment and the committee medical assessment (Pearson’s correlation r = 0.80, p < 0.001)^12^.

We defined cases more than IIa as dementia development, as validated by a previous study^13^. The standardized assessment manual for the cognitive disability in older people defines IIa as “individuals who had dementia-related symptoms, behavioural disturbance and/or difficulty in communication that limited daily living outside the home, but who were capable of daily living under someone’s care”^8^.

### Definition of regular walking

We assessed average walking time per a day (<30, 30–59, 60–89, and ≥90 minutes), and we defined walking time per a day ≥30 minutes as regular walking.

### Statistical analysis

We calculated person-months of follow-up from baseline to the first endpoint: dementia, death, moving away from the local government area where they were registered, loss to follow-up, or administrative censoring at February 21, 2017.

Multivariable adjusted hazard ratios (HRs) with 95% confidence intervals (CIs) for dementia development according to existence of knee/low back pain were calculated using a Cox regression model. We also reran the main model after stratifying participants by age group or regular walking. As a sensitivity analysis, we reran the models after excluding participants suspected of mood or anxiety disorder (K6 ≥ 13 points) and those with both knee and low back pain.

P-values < 0.05 (two-tailed tests) were considered statistically significant. All statistical analyses were performed using SAS version 9.4 (SAS Institute Inc., Cary, NC, USA).

### Statement of ethics

All procedures were in accordance with the ethical standards of the Helsinki Declaration of 1975, as revised in 2013. All respondents were regarded as having provided their informed consent by returning the questionnaire. The Japan Gerontological Evaluation Study was approved by the Nihon Hukushi University Institutional Review Boards on human research (No. 13–14; date of approval: August 6, 2013). The title of the research was “The Japan Gerontological Evaluation Study (JAGES) project: the population-based large cohort.”

## Results

Table 1 shows the mean values and proportions for participant characteristics according to existence of knee pain or low back pain, and according to no pain, knee pain only, low back pain only, and both knee pain and low back pain during the past year. Compared with participants without knee pain or low back pain, those with knee pain or low back pain were more likely to be obese; to have high blood pressure, low income, and mood or anxiety disorder; to have lost a partner, relative, family, or friends in the last year; to go out less than once a week; and were less likely to be single, meet friends or acquaintances, and walk regularly.

**Table 1:** Mean values and proportions for participant characteristics.

	Knee pain		Low back pain		No pain	Knee pain only	Low back pain only	Both knee and low back pain
	−	+	−	+				
Number	10785	3842	10375	4252	8964	1411	1821	2431
Age, year (SD)	72.8 (5.8)	74.9 (6.3)	73.0 (5.8)	74.1 (6.3)	72.8 (5.7)	74.6 (6.2)	72.8 (6.0)	75.1 (6.4)
	n (%)	n (%)	n (%)	n (%)	n (%)	n (%)	n (%)	n (%)
Women	5339 (49.5)	2444 (63.6)	5371 (51.8)	2412 (56.7)	4447 (49.6)	924 (65.5)	892, (49.0)	1520 (62.5)
Obese	2130 (19.8)	1194 (31.1)	2181 (21.0)	1143 (26.9)	1742 (19.4)	439 (31.1)	388, (21.3)	755 (31.1)
Drinker	4083 (37.9)	1143 (29.8)	3800 (36.6)	1426 (33.5)	3392 (37.8)	408 (28.9)	691, (37.9)	735 (30.2)
Current smoker	1195 (11.1)	324 (8.4)	1096 (10.6)	423 (1.0)	988 (11.0)	108 (7.7)	207, (11.4)	216 (8.9)
History of diabetes mellitus	1331 (12.3)	540 (14.1)	1298 (12.5)	573 (13.5)	1112 (12.4)	186 (13.2)	219, (12.0)	354 (14.6)
History of hyper tension	4934 (45.8)	2082 (54.2)	4799 (46.3)	2217 (52.1)	4059 (45.3)	740 (52.5)	875, (48.1)	1089 (55.2)
Did not graduate from high school	4001 (37.1)	1881 (49.0)	3969 (38.3)	1913 (45.0)	3337 (37.2)	632 (44.8)	664, (36.5)	1249 (51.4)
Single	250 (2.3)	53 (1.4)	232 (2.2)	71 (1.7)	217 (2.4)	15 (1.1)	33, (1.8)	38 (1.6)
Low income	2725 (25.3)	1404 (36.5)	2681 (25.8)	1448 (34.1)	2235 (24.9)	446 (31.6)	490, (26.9)	958 (39.4)
Retired, n (%)	6428 (59.6)	2177 (56.7)	6152 (59.3)	2453 (57.7)	5320 (59.4)	832 (59.0)	1108 (60.9)	1345 (55.3)
Lost partner in last year	1224 (11.4)	659 (17.2)	1109 (10.7)	774 (18.2)	927 (10.3)	182 (12.9)	297, (16.3)	477 (19.6)
Lost relative, family, or friends in last year	3397 (31.5)	1331 (34.6)	3238 (31.2)	1490 (35.0)	2765 (30.9)	473 (33.5)	632, (34.7)	858 (35.3)
Frequency of going out <1/week	247 (2.3)	162 (4.3)	235 (2.3)	176 (4.1)	191 (2.1)	44 (3.1)	56, (3.1)	120 (4.9)
Never meets friends or acquaintances	695 (6.4)	335 (8.7)	686 (6.6)	344 (8.1)	577 (6.4)	109 (7.7)	118, (6.5)	226 (9.3)
Mood or anxiety disorder	353 (3.3)	312 (8.1)	339 (3.3)	326 (7.7)	263 (2.9)	76 (5.4)	90, (4.9)	236 (9.7)
Knee pain	—	—	1411 (13.6)	2431 (57.2)	—	—	—	—
Low back pain	1821 (16.9)	2431 (63.3)	—	—	—	—	—	—
Lack of regular walking	2243 (20.8)	1138 (29.6)	2129 (20.5)	1252 (29.4)	1770 (19.8)	359 (25.4)	473, (26.0)	779 (32.0)

Abbreviations: SD: standard deviation. Note: Definition of obese: body mass index ≥25.

In total, 482 (3.3%) developed dementia during follow-up. Table 2 shows the multivariable HRs for dementia development according to existence of knee or low back pain. Compared with participants without knee pain, those with knee pain had increased risk of dementia development [Model 6, HR 1.32 (95% CI: 1.06–1.64)].

**Table 2:** Hazard ratios for dementia development according to knee pain and low back pain.

	Knee pain		Low back pain	
	−	+	−	+
**Total**				
Person months	1008047.1	354044.7	96819.8	393922.0
Number of cases	298	184	322	160
Model 1 HR (95% CI)	1	1.77 (1.47–2.12)^‡^	1	1.22 (1.01–1.48)*
Model 2 HR (95% CI)	1	1.77 (1.47–2.13)^‡^	1	1.22 (1.01–1.47)*
Model 3 HR (95% CI)	1	1.21 (1.00–1.46)*	1	0.96 (0.79–1.16)
Model 4 HR (95% CI)	1	1.31 (1.06–1.62)*	1	0.85 (0.68–1.05)
Model 5 HR (95% CI)	1	1.39 (1.12–1.72)^†^	1	0.84 (0.68–1.05)
Model 6 HR (95% CI)	1	1.32 (1.06–1.64)*	1	0.79 (0.63–0.99)*

Model 1: Crude model.

Model 2: Adjusted for sex.

Model 3: Adjusted for sex and age.

Model 4: Adjusted for above-mentioned variables plus knee pain or low back pain.

Model 5: Adjusted for above-mentioned variables plus body mass index, alcohol consumption, smoking, history of diabetes mellitus, and history of hyper tension.

Model 6: Adjusted for above-mentioned variables plus education, marital status, equivalised income, employment status, loss events, frequency of going out, frequency of social interaction and mood or anxiety disorder.

Abbreviations: HR: hazard ratio, CI: confidence interval. *p < 0.05, **p < 0.01.

In contrast, those with low back pain had decreased risk of dementia development compared with those without low back pain [Model 6, HR 0.79 (95% CI: 0.63–0.99)]. In model 1 and model 2 (the crude model and the sex-adjusted model), those with low back pain had an increased risk of dementia development. However, after adjusting for age, those with low back pain had a decreased risk of dementia development compared with those without low back pain. Therefore, we calculated HRs of dementia stratified by age group (Table 3). Both knee pain and low back pain increased the dementia risk among individuals aged ≤79 years, and decreased the dementia risk among individuals aged ≥80 years after adjusting for knee pain and low back pain.

**Table 3:** Hazard ratios of dementia stratified by age group.

	p for interaction	Knee pain		p for interaction	Low back pain	
		−	+		−	+
**65**–**69 aged years**						
Person months	—	345697.9	80076.4	—	315241.7	110532.7
Number of cases	—	30	4	—	29	5
Model 2 HR (95% CI)	—	1	N/A	—	1	N/A
Model 7 HR (95% CI)	—	1	N/A	—	1	N/A
**70**–**74 aged years**						
Person months	—	329330.5	105478.5	—	318097.8	116711.2
Number of cases	—	38	25	—	41	22
Model 2 HR (95% CI)	p = 0.01	1	2.09 (1.26–3.49)^†^	p = 0.02	1	1.47 (0.88–2.47)
Model 7 HR (95% CI)	p = 0.02	1	2.04 (1.14–3.64)*	p = 0.02	1	1.41 (0.93–2.15)
**75**–**79 aged years**						
Person months	—	199995.7	86935.5	—	199571.9	87359.30
Number of cases	—	53	39	—	57	35
Model 2 HR (95% CI)	—	1	1.72 (1.13–2.62)*	—	1	1.41 (0.92–2.15)
Model 7 HR (95% CI)	—	1	1.63 (1.01–2.64)*	—	1	1.11 (0.68–1.80)
**80**–**84 aged years**						
Person months	—	94293.8	55026.1	—	95807.3	53512.60
Number of cases	—	91	51	—	95	47
Model 2 HR (95% CI)	—	1	0.93 (0.66–1.32)	—	1	0.87 (0.62–1.24)
Model 7 HR (95% CI)	—	1	0.99 (0.67–1.47)	—	1	0.88 (0.59–1.30)
**85**–**89 aged years**						
Person months	—	31497.3	20941.6	—	32105.7	20333.30
Number of cases	—	59	44	—	72	31
Model 2 HR (95% CI)	—	1	1.11 (0.75–1.65)	—	1	0.67 (0.44–1.03)
Model 7 HR (95% CI)	—	1	1.45 (0.94–2.25)	—	1	0.56 (0.35–0.90)*
**≥90 aged years**						
Person months	—	7231.8	5586.7	—	7345.5	5473.00
Number of cases	—	27	21	—	28	20
Model 2 HR (95% CI)	—	1	1.15 (0.64–2.09)	—	1	1.03 (0.58–1.84)
Model 7 HR (95% CI)	—	1	1.18 (0.60–2.33)	—	1	0.95 (0.49–1.85)

Model 2: Adjusted for sex.

Model 7: Adjusted for sex and knee pain or low back pain.

Abbreviations: HR: hazard ratio, CI: confidence interval. *p < 0.05, **p < 0.01.

Table 4 shows the multivariable HRs for dementia development according to no pain, knee pain only, low back pain only, and both knee pain and low back pain during the past year stratified by age group; 65–79 and ≥80 years old. Participants with knee pain only aged 65–79 years had an increased risk of dementia development [Model 9, HR 1.73 (95% CI: 1.11–2.68)]. Those with low back pain aged ≥80 years had a decreased risk of dementia development [Model 9, HR 0.50 (95% CI: 0.31–0.80)].

**Table 4:** Hazard ratios of dementia development according to no pain, knee pain only, low back pain only, and both knee pain and low back pain during the past year stratified by age group.

	No pain	Knee pain only	Low back pain only	Both knee and low back pain
**Total**				
Person months	837564.4	130605.4	170482.7	223439.3
Number of case	258	64	40	120
Model 1 HR (95% CI)	1	1.60 (1.21–2.10)^‡^	0.76 (0.55–1.06)	1.75 (1.41–2.17)^‡^
Model 2 HR (95% CI)	1	1.60 (1.21–2.11)^‡^	0.76 (0.55–1.06)	1.75 (1.41–2.17)^‡^
Model 3 HR (95% CI)	1	1.16 (0.88–1.53)	0.71 (0.51–0.99)*	1.14 (0.91–1.42)
Model 8 HR (95% CI)	1	1.25 (0.95–1.65)	0.72 (0.52–1.02)	1.20 (0.96–1.50)
Model 9 HR (95% CI)	1	1.20 (0.91–1.59)	0.69 (0.50–0.97)*	1.07 (0.84–1.34)
**65**–**79 aged years**				
Person months	729847.4	103063.9	14516.7	169426.4
Number of case	100	27	21	41
Model 9 HR (95% CI)	1	1.73 (1.11–2.68)*	1.04 (0.65–1.67)	1.39 (0.94–2.05)
**≥80 aged years**				
Person months	107717	27541.5	25306	54012.8
Number of case	200	52	30	103
Model 9 HR (95% CI)	1	0.94 (0.65–1.35)	0.50 (0.31–0.80)^†^	0.91 (0.68–1.22)

Model 1: Crude.

Model 2: Adjusted for sex.

Model 3: Adjusted for sex and age.

Model 8: Adjusted for above-mentioned variables plus body mass index, alcohol consumption, smoking, history of diabetes mellitus, and history of hyper tension.

Model 9: Adjusted for above-mentioned variables plus education, marital status, equivalised income, employment status, loss events, frequency of going out, frequency of social interaction, and mood or anxiety disorder.

Abbreviations: HR, hazard ratio; CI, confidence interval. *p < 0.05, ^‡^p < 0.001.

Table 5 shows the multivariable HRs for dementia development stratified by regular walking. The associations of knee pain and low back pain with dementia risk were similar to the main results, regardless of regular walking. Individuals aged 65–79 years and ≥80 years who did not walk regularly and had knee pain had the highest dementia risk of the four groups (with/without knee pain; regular walking/no regular walking) [Model 6, HR: 1.91 (95% CI: 1.16–3.16), and HR: 1.63 (95% CI: 1.10–2.43), respectively]. Participants who experienced low back pain and did not walk regularly did not have an increased risk of dementia development compared with those with no low back pain who walked regularly.

**Table 5:** Hazard ratios of dementia stratified by regular walking.

		p for interaction	Knee pain		p for interaction	Low back pain	
			−	+		−	+
Regular walking	Person months of total		800788.7	250171.0		771816.7	279142.9
	Number of case		192	106		211	87
	Model 6 HR (95% CI)	0.52	1	1.38 (1.06–1.80)*	0.51	1	0.81 (0.62–1.07)
Lack of regular walking	Person months of total		207258.4	103873.7		196353.1	114779.1
	Number of case		106	78		111	73
	Model 6 HR (95% CI)	—	1.40 (1.10–1.80)^†^	1.71 (1.26–2.33)^‡^	—	1.40 (1.10–1.78)^†^	1.00 (0.74–1.34)
**65–79 aged years**							
Regular walking	Person months of total		704269.7	196443.2		672304.2	228408.8
	Number of case		85	41		89	37
	Model 6 HR (95% CI)	0.78	1	1.60 (1.06–2.43)*	0.60	1	0.96 (0.63–1.47)
Lack of regular walking	Person months of total		170754.3	76047.3		160607.2	86194.4
	Number of case		36	27		38	25
	Model 6 HR (95% CI)	—	1.31 (0.87–1.96)	1.91 (1.16–3.16)*	—	1.34 (0.91–1.99)	1.09 (0.66–1.79)
**≥80 aged years**							
Regular walking	Person months of total		96518.9	53727.8		99512.6	50734.2
	Number of case		107	65		122	50
	Model 6 HR (95% CI)	0.71	1	1.25 (0.88–1.75)	0.80	1	0.72 (0.50–1.04)
Lack of regular walking	Person months of total		36504.1	27826.5		35745.9	28584.7
	Number of case		70	51		73	48
	Model 6 HR (95% CI)	—	1.44 (1.05–1.98)*	1.63 (1.10–2.43)*	—	1.42 (1.04–1.94)*	0.96 (0.66–1.40)

Model 6: Adjusted for age, sex, body mass index, knee pain or low back pain, alcohol consumption, smoking, history of diabetes mellitus, history of hyper tension, education, marital status, equivalised income, employment status, loss events, frequency of going out, frequency of social interaction, and mood or anxiety disorder.

Abbreviations: HR, hazard ratio; CI, confidence interval. *p < 0.05, ^†^p < 0.01, ^‡^p < 0.001.

The sensitivity analysis showed similar results to the main results (data not shown).

## Discussion

In this sample of the Japanese general population, knee pain was prospectively associated with increased risk of dementia development particularly in individuals aged 65–79 years. The increased dementia risk in these individuals may be enhanced if they do not walk regularly. Low back pain was associated with reduced risk of dementia development among individuals ≥80 aged years, independent of physical activity, socioeconomic and psychosocial factors.

Knee pain among older people, which is usually caused by OA, is associated with inflammation^14,15^. Persistent inflammation damages cerebral blood vessels and induces neuroinflammation^14,15^. Research shows that both vascular dementia and Alzheimer’s dementia are caused by inflammation^14,15^. Individuals with knee pain are likely to have a high sedative load, which can increase dementia risk. However, we suggest that knee pain (possibly accompanied by inflammation) itself, as well as physical inactivity, might increase dementia risk. Inflammation also contributes to some subtypes of low back pain, but the prevalence of inflammatory low back pain in one population-based study was only 5–6%^16^.

A previous cohort study in Taiwan reported an association between OA and dementia development^4^. However, this previous observational study had several limitations. First, it used data from a health insurance database based on physicians’ diagnoses in medical settings, and included only patients visiting hospitals or clinics; thus, the study did not consider individuals who had knee pain but did not consult a doctor. Second, this previous study did not account for socioeconomic and psychosocial factors in addition to physical activity; all of these are important risk factors for dementia. The present study did not have these limitations.

Approximately 85% of low back pain is a non-specific type of pain related to central nerve system^17^. A systematic literature review reported that OA pain including knee pain was also associated with central nerve system^18^. For a person to perceive a centralized pain, it is necessary for the prefrontal brain area to pay “sustained attention” to the pain^19^, and sustained attention requires maintaining a certain level of brain function. For example, patients with cognitive disorder owing to dementia or Parkinson’s disease often have decreased sustained attention^20,21^. Participants with low back pain aged ≥80 years had a decreased risk of dementia compared with those without low back pain. These findings may suggest that experiencing low back pain may be a marker of relatively maintained brain function among old-old people. We found that participants aged 65–79 years with knee pain only had an increased risk of dementia, but those aged ≥80 years did not. Although inflammation related to knee pain may increase dementia risk, experiencing knee pain may be associated with maintained brain function particularly for old-old people; thus, old-old people (unlike other generations) may not have an increased risk of dementia.

One of the strengths of the present study is that we used a community-based prospective large cohort study design. However, the study has several limitations. First, data on the development of dementia were obtained from the results of an examination and assessment for a national Long-Term Care scheme. Residents aged ≥40 years with specific diseases and all residents aged ≥65 years can apply for the long-term care program if they wish; participants with dementia development who did not apply for the national long-term care program were not identified. Therefore, the number of individuals with dementia development might have been underestimated. Second, we were unable to differentiate dementia type (e.g., vascular or Alzheimer’s dementia). Analysis of pathological information may have elucidated the mechanisms underlying the associations found here. Third, we did not measure pain intensity and therefore could not examine dose–response relationships between low back and knee pain and dementia risk. Fourth, we did not collect information about medical treatments. Treatment for pain, dementia, and other comorbidities may affect dementia development; however, we could not examine this potential confounding effect.

In conclusion, knee pain may increase dementia risk among Japanese older people aged 65–79 years, and this increased dementia risk may be enhanced if they do not walk regularly, independent of socioeconimic and psychosocial factors. Experience of low back pain may be a marker of maintained cognitive function despite age in old-old people aged ≥80 years, independent of physical activity, socioeconomic and psychosocial factors.

## Data Availability

All enquiries should be addressed to the data management committee via e-mail: dataadmin.ml@jages.net. All JAGES datasets have ethical or legal restrictions for public deposition owing to inclusion of sensitive information from the human participants. Following the regulation of the local governments that cooperated with our survey, the JAGES data management committee has imposed restrictions upon the data.
